# *EbMYBP1*, a R2R3-MYB transcription factor, promotes flavonoid biosynthesis in *Erigeron breviscapus*

**DOI:** 10.3389/fpls.2022.946827

**Published:** 2022-07-28

**Authors:** Yan Zhao, Guanghui Zhang, Qingyan Tang, Wanling Song, Qingqing Gao, Guisheng Xiang, Xia Li, Guanze Liu, Wei Fan, Xiaoning Li, Shengchao Yang, Chenxi Zhai

**Affiliations:** ^1^Key Laboratory of Medicinal Plant Biology of Yunnan Province, National and Local Joint Engineering Research Center on Germplasms Innovation and Utilization of Chinese Medicinal Materials in Southwest China, Yunnan Agricultural University, Kunming, China; ^2^College of Agronomy & Biotechnology, Yunnan Agricultural University, Kunming, China; ^3^Sibley School of Mechanical and Aerospace Engineering, Cornell University, Ithaca, NY, United States

**Keywords:** *Erigeron breviscapus*, MYB transcription factor, flavonoid biosynthesis, secondary metabolism, scutellarin

## Abstract

*Erigeron breviscapus*, a traditional Chinese medicinal plant, is enriched in flavonoids that are beneficial to human health. While we know that R2R3-MYB transcription factors (TFs) are crucial to flavonoid pathway, the transcriptional regulation of flavonoid biosynthesis in *E. breviscapus* has not been fully elucidated. Here, *EbMYBP1*, a R2R3-MYB transcription factor, was uncovered as a regulator involved in the regulation of flavonoid accumulation. Transcriptome and metabolome analysis revealed that a large group of genes related to flavonoid biosynthesis were significantly changed, accompanied by significantly increased concentrations of the flavonoid in *EbMYBP1-*OE transgenic tobacco compared with the wild-type (WT). *In vitro* and *in vivo* investigations showed that *EbMYBP1* participated in flavonoid biosynthesis, acting as a nucleus-localized transcriptional activator and activating the transcription of flavonoid-associated genes like *FLS*, *F3H*, *CHS*, and *CHI* by directly binding to their promoters. Collectively, these new findings are advancing our understanding of the transcriptional regulation that modulates the flavonoid biosynthesis.

## Introduction

Flavonoids are one of the most abundant secondary metabolites and are found in plants across a wide range of morphological classes (Buer et al., 2010; Tohge et al., 2013). There are several main classes of derivatives produced by the phenylpropanoid pathway, such as flavonols, anthocyanins, and proanthocyanidins (Routaboul et al., 2012; Saito et al., 2013). Flavonols have excellent antioxidant properties and are thought to perform key functions as UV filters, exhibiting important roles during plant evolution (Pollastri and Tattini, 2011). Additionally, flavonoids are known to exhibit anti-inflammatory, anti-proliferative, and antioxidative properties, preventive properties toward cardiovascular disease as well as diabetes (Williams et al., 2004; Perez-Vizcaino and Duarte, 2010; Zhang et al., 2015; Zakaryan et al., 2017).

Flavonoids are synthesized through the phenylpropanoid pathway in plants, initially catalyzed by phenylalanine ammonia lyase (*PAL*), cinnamate 4-hydroxylase (*C4H*) and 4-hydroxy-cinnamoyl CoA ligase (*4CL*) (Ferreyra et al., 2021). Great progress has been made to elucidate the structural genes of the flavonoid biosynthesis pathway. It was found that *CHS* catalyzes the first step in flavonoid biosynthesis, converting *p*-coumaryl-CoA and malonyl-CoA to naringenin chalcone (Pandith et al., 2016). *CHS* further converts the initial product into many flavonoids, including flavonol, flavone, flavanone and anthocyanidin. Another enzyme, flavonol synthase (*FLS*), a key enzyme in the flavonol pathway, contributes to the synthesis of flavonols. It competes with dihydroflavonol 4-reductase (*DFR*) for the same substrate, dihydroflavonol (Wellmann et al., 2002; Davies and Schwinn, 2003; Luo et al., 2008; Sheng et al., 2020). In sum, numerous regulatory proteins including transcription factors (TFs) are associated with flavonoid biosynthesis.

Flavonoid biosynthesis has been studied in many plant species. A wide variety of transcription factors (TFs) are implicated in flavonoid biosynthesis, including MYB, basic helix-loop-helix (bHLH), basic leucine zipper (bZIP), WD40, and zinc finger proteins (Chen et al., 2019; Naik et al., 2022). In plants, MYB TFs control the transcription of flavonoid biosynthesis genes, a process which has been extensively studied. MYB genes are a major transcription factor family in plants and are involved in various biological processes. MYB proteins, including MYB12, MYB11, and MYB111, have been identified in *Arabidopsis* as flavonol-specific factors based on sequence similarity (Mehrtens et al., 2005; Stracke et al., 2007). Furthermore, the SG7 MYBs are found in grapes (Czemmel et al., 2009; Matus et al., 2009), apples (Wang et al., 2017), pears (Zhai et al., 2019; Premathilake et al., 2020), peaches (Cao et al., 2019) and other plants (Ballester et al., 2010; Liu et al., 2016). Additionally, *AgMYB12*, located in the S7 subgroup of the R2R3-MYB family, displayed as a positive regulator of apigenin biosynthesis, activating the expression of *AgFNS* gene in celery (Wang et al., 2022). These results showed that MYB TFs play key regulatory roles in flavonoid biosynthesis.

*Erigeron breviscapus* (*E. breviscapus*) is a medicinal plant in the Compositae family with great medicinal potential, comprised of 25 kinds of flavonoids, 46 kinds of caffeoyl compounds, 78 kinds of volatile oils, and nearly 40 kinds of other compounds (Wu et al., 2021). Previously, a total of 108 R2R3-MYB transcription factors have been identified in the *E. breviscapus* genome (He et al., 2021; Song et al., 2021). Phylogenetic analysis suggests possible involvement of several MYBs in phenylpropanoid metabolism including flavonoids, flavonols, and anthocyanins (Song et al., 2021). However, no transcription factor involved in flavonoid biosynthesis has been identified in *E. breviscapus*. Thus, it is imperative to identify MYB TFs in *E. breviscapus* that can specifically and positively regulate flavonoid biosynthesis, the findings for which will provide us with a greater understanding of the flavonoid biosynthesis pathway. Here, *EbMYBP1*, a flavonoid biosynthesis regulator, was isolated from *E. breviscapus.* Transcriptome and metabolome analysis showed that overexpression of *EbMYBP1* resulted in an increased flavonoid content in transgenic tobacco lines through upregulation of flavonoid-related biosynthesis genes *FLS*, *F3H*, *CHS*, and *CHI*. Our finding will provide valuable insights to elucidate the roles of *EbMYBP1* in positively regulating flavonoid biosynthesis in *E. breviscapus*.

## Materials and methods

### Plant materials

In this study, *Erigeron breviscapus cv.* Long Jin NO. 1 (LJ1) and *Nicotiana tabacum cv. Yunyan87* (Y87) were used as plant materials, which were obtained from Longjin Biotech Co., Ltd. (Xuanwei, Yunnan, China) and Yunnan Agricultural University (Kunming, Yunnan, China), respectively. Seeds of LJ1 were sowed in nutrient soils and grown in a greenhouse in Yunnan Agricultural University. Seeds of Y87 were surface sterilized with 75% alcohol for 30 s, 10% H_2_O_2_ for 10 min, washing five times with sterile H_2_O, and placing the seeds on MS medium for 2–3 weeks. Sterilized plants were prepared for use in transgenic plants.

### Measurement of scutellarin content

The roots, stems, leaves, flowers samples of *E. breviscapus* were classified, and three biological replicates were set for each tissue. After dried at a constant temperature at 45°C, the samples were ground into a fine powder using a high-speed grinder (MM400, Zhongxingwy, Beijing, China). Then, 30 mL of 80% methanol was added into 1 g of root, stem, leaf, flower powder. To obtain the filtrate, the sample was kept at 4°C overnight, then centrifuged for 12 min at 6,000 × g. Extracted samples were analyzed using UPLC (UPLC, Agilent 1260 Infinity). The analysis was performed using a binary mobile phase composed of A: water + 0.1% phosphoric acid and B: 100% acetonitrile. The following conditions were used in the analysis: UPLC column, SunFire C18 (5 μm, 4.6 × 250 mm); mobile phase solvent A, water with 0.1% phosphoric acid, solvent B acetonitrile with 0.1% phosphoric acid. The following gradient conditions were used to achieve separation: starting at 80% A at 0 min, 80% A at 3 min, 74.5% A at 17 min, 56% A at 28 min, 38% A at 30 min, 38% A at 32 min and holding for 5 min. The flow rate was 1 mL/min, the column temperature was 40°C, and the injection volume was 8 μL.

### Molecular cloning and characterization of *EbMYBP1*

Total genomic DNA and RNA were isolated from the leaf tissues of *E. breviscapus* using a DNA/RNA isolation kit (TIANGEN, China). Based on the manufacturer’s instructions, cDNA was synthesized using a PrimeScript RT Reagent Kit with gDNA Eraser (Takara, Japan). Amplification of full length *EbMYBP1* cDNA with gene specific primers (see Supplementary Table 1) was performed by PCR, after which the sequence was generated. The generated sequence was checked against GenBank by BLASTX and BLASTp. Amino acid sequence alignments were performed by ClustalX, and a phylogenetic tree of EbMYBP1 and of closely related proteins in other plant species was constructed by MEGA 7.0.

### Subcellular localization of *EbMYBP1*

The full-length cDNA sequence of EbMYBP1 was inserted into pCAMBIA1300-35S-N-GFP and pCAMBI A1300-35S-GFP-C vectors, resulting in pCAMBIA1300-35S-EbMYBP1-GFP (EbMYBP1-GFP) and pCAMBIA1300-35S-GFP-EbMYBP1 (GFP-EbMYBP1) fusion constructs, which were subsequently transformed into *A. tumefaciens* GV3101 by the electroporation method. The pCAMBIA1300-35S-N-GFP and pCAMBIA1300-35S-GFP-C vectors were used as controls. *Agrobacterium* was cultured on YEB agar supplemented with selection antibiotics, and then incubated at 28°C for 2∼3 day. The confluent *Agrobacterium* containing the target vector was resuspended in an infiltration buffer (0.5 × MS, 10 mM MES (pH 5.6), 150 μg/mL acetosyringone) to an OD_600_ of 0.5 and incubated at room temperature without shaking for 2 h before infiltration. Approximately 500 μL of the *Agrobacterium* mixture was then infiltrated into 3–4 young leaves of each plant, with at least two points for each leaf. The subcellular localization assay was performed 48 h after inoculation. Confocal images were taken by using a Zeiss LSM 880 confocal laser scanning microscope (Zeiss, Germany).

### Transcriptional activity assay

The ORF sequence of EbMYBP1 was amplified and ligated into pGBKT7 containing a GAL4 DNA-binding domain, generating the pGBKT7-EbMYBP1. The pGBKT7-EbMYBP1, negative control (pGBKT7 or pGADT7), and positive control (pGADT7 + pGBKT7-53) vectors were transformed into Y2HGold yeast cells. The yeast cells were cultivated on SD/-Trp medium or SD–Trp/His/Ade medium. Transcriptional activity was assayed by the growth status. The primers used are listed in Supplementary Table 1.

### Tobacco transformation

The CDS of *EbMYBP1* was cloned from leaves of 8-week-old *E. breviscapus.* The target fragments were subcloned into the PC1300-35S vector between the *Sma*I and *Xba*I sites to generate a PC1300-35S-*EbMYBP1* construct using the primers listed in Supplementary Table 1. Then, the plasmids were introduced into *Agrobacterium tumefaciens* GV3101, which was then used in tobacco cultivar Yunyan87 (*Nicotiana tabacum*) transformation by the leaf disc methods. Regenerated shoots and healthy resistant shoots were grown on selective shooting medium and rooting medium, respectively, which both contained 50 mg⋅L^–1^ kanamycin and 250 mg⋅L^–1^ carbenicillin. Well-developed rooted plants were transferred to soil and then grown in a growth room at 25 ± 2°C, with 65–70% relative humidity.

Leaves were harvested from 7-week-old plants. The samples from Yunyan87 (wild-type, WT) and homozygous T2 lines of transgenic tobacco plants (*EbMYBP1-OE*), were collected for transcriptome and metabolome sequencing. All samples were frozen immediately in liquid nitrogen and stored at −80°C. The leaves from six individual plants were sampled as one biological replicate, and three biological replicates were used in this study.

### Measurement of total flavonoids content

The leaves of tobacco from six different plants that grew for about 7 weeks were collected to quantify the total flavonoids content. Flavonoids were extracted and quantified using the method (Wang et al., 2016). The absorbance of the samples was determined at 535 nm with a spectrophotometer (UV-1800, Shimadzu), and methanol with 1% HCl was used as a blank control. The total flavonoids content was measured with (mg/g FW) = (1/958 × A_535_ × 10,000 × V)/fresh weight (g), where the V indicates the total volume of the extract (mL). At least three biological replicates were used for each sample.

### Metabolic analysis

Metabolite profiling was performed using an LC–ESI–MS/MS system (UPLC; Shim-pack UFLC SHIMADZU CBM30A, Shimadzu, Kyoto, Japan; MS/MS, Applied Biosystems 6500 QTRAP, Applied Biosystems, Foster City, CA, United States) (Chen et al., 2013). The data from six samples (*EbMYBP1*-OE and WT × three biological replicates) were processed by OPLS-DA and PCA to detect differences in metabolic composition between the *EbMYBP1*-OE and WT tobacco.

### Transcriptome analysis

The seven-week-old tobacco leaves of the WT and *EbMYBP1-OE* were used for transcriptome analysis, and three biological replicates were made for each sample. Firstly, RNA was prepared, and reverse transcribed into the cDNA library. In order to ensure the quality of the data, fastp was used to control the raw data. 1% agarose gel, Nanophotometer spectrophotometer and Agilent 2100 Bioanalyzer were used to analyze the purity of the RNA and detect the integrity of the RNA. These data were subsequently used to analyze base composition and mass distribution to confirm the accuracy of this set of data. We used HISAT2 software for alignment analysis with the reference genome, assembled the transcripts of the new genes with Stringtie, analyzed the gene expression level with FPKM, screened the significantly different accumulation genes according to FDR < 0.05 and fold change ≥ 2 with DESeq2, and enriched GO and KEGG with Tbtools software (Chen et al., 2020) and the KEGG database. The transcriptome data of WT and *EbMYBP1-OE* have been deposited to national center for biotechnology information database as a sequence read archive under BioProject ID (PRJNA836017).

### Dual-luciferase reporter assays

The instantaneous color determination experiment was performed on the leaves of 4-week-old tobacco seedlings. The promoter regions of tobacco cultivar Yunyan87 (*Nicotiana tabacum*) genes *NtFLS2*, *NtF3H*, *NtFLS1*, *NtDFR*, *NtCHI*, and *NtCHS* were amplified and cloned into pGreenII 0800-LUC vector making *pGreen 0800:FLS2, pGreen 0800:F3H, pGreen 0800:FLS1, pGreen 0800:DFR, pGreen 0800:CHI, and pGreen 0800:CHS* reporter constructs. The *EbMYBP1* ORF was amplified and cloned into the *pGreenII 62-*SK vector to obtain the *35S:EbMYBP1* effector construct. LUC and REN luciferase activities were determined using a dual luciferase assay kit (Promega, United States) according to the manufacturer’s instructions. These plasmids were transiently infiltrated in tobacco leaves by *Agrobacterium*. Three biological repeats were included in each combination.

### EMSA analysis

The ORF sequence of *EbMYBP1* was cloned and fused with the GST tag in pGEX-4T-1 vectors (GE Healthcare Life Science) and expressed in *Escherichia coli* strain Rosetta (DE3) by induction with 0.5 mM isopropyl-β-D-thiogalactopyranoside for 6 h at 20°C. The recombinant proteins were purified with a GST-tagged protein purification kit (Clontech). Probes containing MYB-specific *cis*-elements derived from the promoters of *FLS*, *CHS, CHI*, and *F3H* genes were labeled with 5′6-FAM (FITC) fluorescent dye. Unlabeled probes were used in competition assays. MYB-specific *cis*-elements within probes were used in mutation assays. Two biological experiments were performed with similar results. All primer sequences used are listed in Supplementary Table 1.

### RT-qPCR analysis

Total RNA of 7-week-old WT and *EbMYBP1*-OE tobacco leaves was extracted using a total RNA small extraction kit (Magen, China). RNA was reverse transcribed into cDNA with a reverse transcription Kit (Takara, China). A 20 μL reaction system was constructed according to qPCR SYBR Green Master Mix (Vazyme, China). PCR was performed on a QuantStudio 5 (ABI) instrument (Thermo Fisher, Singapore). All primers used in this study are listed in Supplementary Table 1. The *NtActin* gene was used as an internal control, thus obtaining true differences in genes of interest-specific expression. Thermo Fisher Expression of genes was used to calculate the relative expression by the 2^–ΔΔCT^ method (Livak and Schmittgen, 2001).

### Statistical analysis

Statistical analysis of data was conducted using one-way analysis of variance, followed by Tukey’s comparison tests, or using Student’s *t*-test in GraphPad Prism 9 software. Differences were considered statistically significant at *P* < 0.05.

## Results

### Isolation and sequence analysis of *EbMYBP1*

**FIGURE 1:**
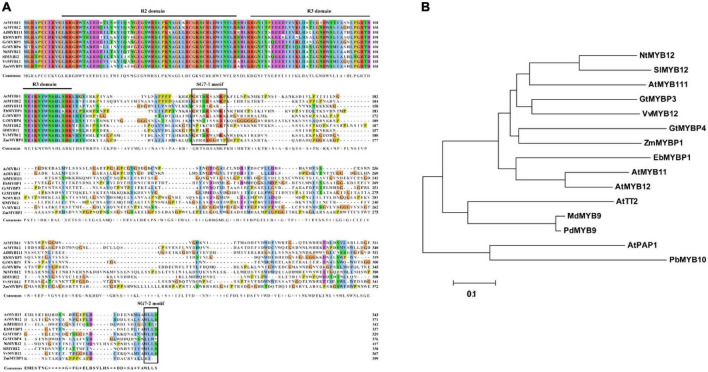
Multiple sequence alignment and phylogenetic relationship of EbMYBP1. **(A)** Multiple sequence alignment of EbMYBP1. The R2/R3 repeats and SG7-1/SG7-2 are indicated in black frame. **(B)** Phylogenetic analysis of EbMYBP1 and other 14 homologs R2R3-MYB proteins. The tree was constructed using MEGA7 software based on the neighbor-jointing method.

The full-length cDNA sequence for *EbMYBP1* was cloned, containing an open reading frame (ORF) of 1,014 bp, encoding 337 amino acid residues, and with a predicted molecular weight of 38.29 kDa. Sequence alignment showed that the R2R3 domains in *EbMYBP1* were highly conserved (Figure 1A). EbMYBP1 contained both SG7 motif-1 ([K/R][R/x][R/K]xGRT[S/x][R/G]xx[M/x]K) and SG7 motif-2 ([W/x][L/x]LS) (Figure 1A), suggesting their putative role in regulating flavonoid biosynthesis.

Next, MYB TFs from different species were selected for phylogenetic analysis. Phylogenetic analysis showed that EbMYBP1 is clustered with some SG7 family proteins, particularly flavonoid regulators such as *Arabidopsis* (*A. thaliana*) AtMYB11, AtMYB12, and AtMYB111; tobacco (*Nicotiana tabacum*) NtMYB12; tomato (*Solanum lycopersicum* L.) SlMYB12; Japanese gentian (*Gentiana triflora*) GtMYBP3, GtMYBP4; grape (*Vitis vinifera*) VvMYB12; and maize (*Zea mays*) ZmMYBP (Figure 1B). EbMYBP1 was most closely related to AtMYB12 and AtMYB11 in *A. thaliana* as shown in the phylogenetic tree.

### *EbMYBP1* expression is associated with flavonoid accumulation in *Erigeron breviscapus*

**FIGURE 2:**
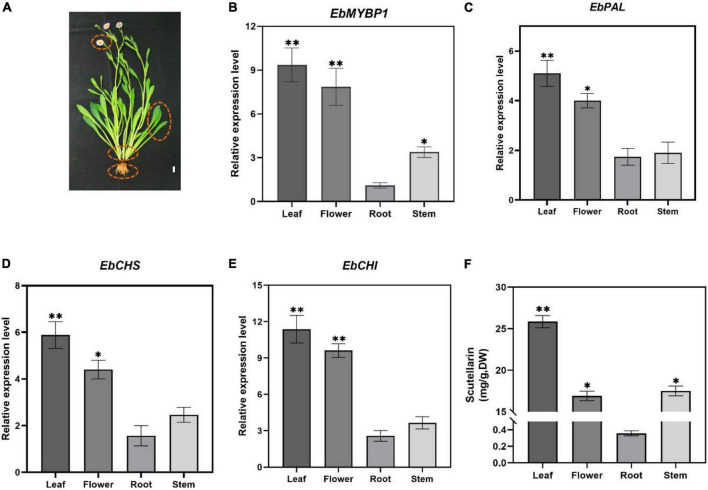
Relative expression of EbMYBP1 in *E. breviscapus*. **(A)** Flowering *E. breviscapus* plants were used in the experiment. Red circles represent flowers, leaves, stems and roots, respectively, which are used in RT-qPCR of *EbMYBP1* and contents of scutellarin analysis, Bar = 1 cm. **(B–E)** Relative expression of *EbMYBP1, EbPAL, EbCHS, and EbCHI* in different tissues of *E. breviscapus*. **(F)** Contents of scutellarin in different tissues of *E. breviscapus*. Data represent mean ± SD of three biological replicates. Asterisks indicate significant differences (*P < 0.05; ^**^P < 0.01).

To determine if *EbMYBP1* expression is associated with flavonoid accumulation in *E. breviscapus*, the expression profiles of *EbMYBP1* in different tissues of *E. breviscapus* were examined. In the transcriptome data of four tissues (flowers, stems, leaves and roots) in our laboratory (Song et al., 2021), *EbMYBP1* was expressed ubiquitously in all four tissues, the highest expression was found in the leaf tissues and the lowest expression was found in the roots by RT-qPCR (Figure 2B). Moreover, the genes in the flavonoid biosynthesis pathway included *EbPAL, EbCHS, and EbCHI*, all of which indicated high expression level in the leaf. Next, we analyzed the flavonoid contents of these four tissues. In all four tissues, four main flavonoids (scutellarin, apigenin-7-O-glucuronide, scutellarein, apigenin) were detected, and their levels varied among tissues (Supplementary Figure 1). Scutellarin accumulation was highest in leaves followed by flowers, stems and roots (Figure 2C). The results showed a positive correlation between *EbMYBP1* expression level and scutellarin accumulation.

### *EbMYBP1* is a nuclear-localized transcriptional activator

**FIGURE 3:**
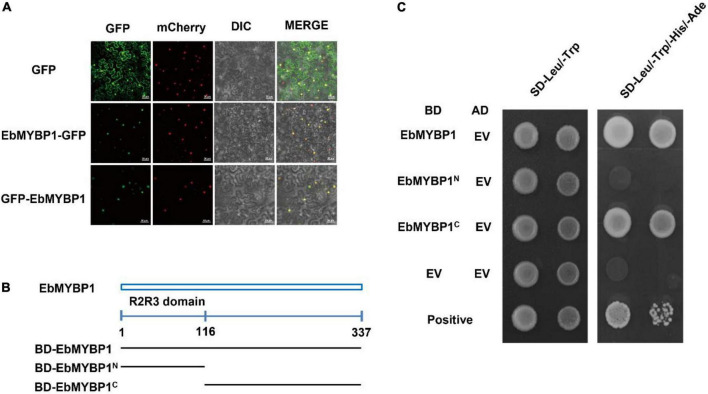
Analysis of the subcellular localization and transactivation activity of EbMYBP1. **(A)** Subcellular localization of EbMYBP1 in *N. benthamiana* leaves. EbMYBP1 fused to the N-terminal of GFP (middle) or C-terminal of GFP (lower) under the control of the 35S promoter. The HY5-mCherry was used as nucleus marker. Differential interference contrast (DIC) and GFP, mCherry fluorescence images as well as merged images are shown. At least three individual experiments were performed for each combination with the similar results. Bars = 50 μm. **(B)** Schematic diagrams illustrate the different portions of EbMYBP1 ORF that were fused to the yeast vector pGBKT7. **(C)** Transactivation activity of the EbMYBP1 protein in yeast. The transformed cells were selected on SD/-Trp medium (left panel) and SD/-Trp/-His medium (right panels). Three biological experiments produce similar results.

To analyze the basic characteristic of EbMYBP1 as a transcription factor, two fusion proteins of EbMYBP1-GFP and GFP-EbMYBP1 were constructed for transient transformation via *Agrobacterium* infiltration methodology. The transient expression results indicated that the fluorescence of EbMYBP1-GFP and GFP-EbMYBP1 was exclusively localized in the nucleus of epidermal cells from young *N. benthamiana* leaves, whereas that of the control GFP protein was distributed throughout the cell (Figure 3A). Thus, the results revealed that EbMYBP1 is a nuclear-localized protein, colocalized in the nucleus.

The transactivation activity of EbMYBP1 was verified in yeast. The complete and various truncated *EbMYBP1* ORFs were cloned into pGBKT7 plasmid, generating GAL4BD- *EbMYBP1* recombinants. The recombinants were transformed into the Y2HGold strain, determining the transactivation ability of EbMYBP1. On the SD/-Trp medium, all the transformants and the negative control pGBKT7 grew well. On the SD-Trp/His/Ade medium, EbMYBP1 containing N-terminal regions did not grow. However, transformants containing the EbMYBP1 C-terminal regions grew well on SD–Trp/His/Ade medium (Figure 3C). These results indicated that EbMYBP1, as reported in the case of several other transcription factors, exhibited transactivation activity and its activation domain is located in the C-terminal region.

### Overexpression of *EbMYBP1* increased flavonoid biosynthesis in transgenic tobacco plants

**FIGURE 4:**
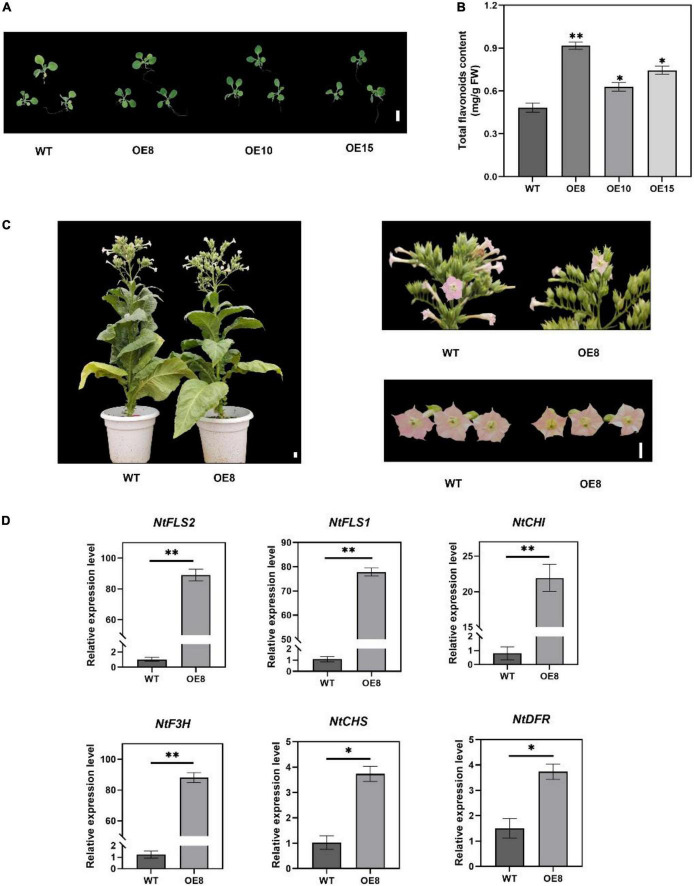
Characterization of transgenic tobacco lines overexpressing *EbMYBP1*. **(A)** Phenotype of transgenic and WT tobacco plants. **(B)** Contents of total flavonoids in the petals of transgenic and WT plants. **(C)** OE8 and WT tobacco plants. **(D)** RT-qPCR assay of the flavonoid pathway genes between WT and OE8. CHS, chalcone synthase; CHI, chalcone isomerase; F3H, flavanone-3-hydroxylase; FLS, flavonol synthase; DFR, dihydroflavonol 4-reductase. Data represent mean ± SD of three biological replicates. Asterisks indicate significant differences (*P < 0.05; ^**^P < 0.01). Bar = 1 cm.

To investigate the possible role of *EbMYBP1* in flavonoid biosynthesis, the complete ORF of *EbMYBP1* was cloned in PC1300-35S to generate the *35S:EbMYBP1* and transformed into tobacco. Three independent transgenic lines *EbMYBP1-*OE-8, *EbMYBP1-*OE-10, and *EbMYBP1-*OE-15 (OE8, OE10, and OE15) were used for phenotype analysis (Supplementary Figure 2). Phenotypic analysis showed no obvious phenotypic changes compared with those of WT plants (Figures 4A,C). To further investigate whether *EbMYBP1* expression is associated with total flavonoid accumulation in tobacco, the *EbMYBP1* expression level and total flavonoid content was analyzed in the leaves of transgenic and WT plants. The total flavonoid content increased significantly by 1.89-, 1.31-, and 1.54- fold in the transgenic lines (OE8, OE10, and OE15 respectively) as compared to that of the WT (*P* < 0.01; Figure 4B). Therefore, OE8 was chosen and used for further transcriptome and metabolome analysis. These results suggested that the *EbMYBP1* expression level was positively related to total flavonoids accumulation.

To further investigate the flavonoid-associated metabolic regulatory network in tobacco overexpressing EbMYBP1, 7-week-old leaves of OE8 were selected for metabolite analysis using UPLC-ESI-MS/MS. PCA analysis was conducted for all samples in WT and OE8. The results showed that these metabolites could be clearly divided into two groups (Supplementary Figure 3). The Partial least squares discriminant analysis (PLS-DA) and Orthogonal projection to latent structures-discriminant analysis (OPLS-DA) model, indicated that the data was reliable and meaningful (Supplementary Figure 4).

A total of 422 metabolites were obtained using a widely targeted metabolomics method (Supplementary Table 2). Furthermore, 98 flavonoid-related metabolites were identified, including 41 flavonols, 29 flavonoids, 7 flavonoid carbonosides, 5 dihydro flavonoids, 4 isoflavones, 4 anthocyanins, 3 dihydroflavonols, 3 flavanols, and 2 chalcones. A clear separation could be observed between WT and OE8, indicating distinct flavonoid profiles in WT and OE8 samples.

Differentially accumulated metabolites (DAMs) were selected according to a fold change ≥2 or ≤0.5 (*P*-value < 0.05). 243 DAMs were identified, and their accumulation was significantly different between WT and OE8, and 27 flavonoids metabolites displayed significantly higher content in the OE8 than in the WT. Moreover, the most prominently differential metabolites were 3,7-Di-O-methylquercetin and kaempferol 3-O-rutinoside in flavonol based on fold change ≥ 2 or ≤ 0.5 and VIP ≥ 1 in the OPLS-DA model (Supplementary Figure 5B and Supplementary Table 3). These results suggest that *EbMYBP1* play an important role in regulation of flavonol biosynthesis.

### Overexpression of *EbMYBP1* upregulated the transcription of flavonoid biosynthesis-associated genes

To investigate the genetic basis of flavonoid-associated metabolism and its relationship to *EbMYBP1* overexpression, RNA-Seq profiling was performed using the leaves of wild-type and *EbMYBP1-*OE (OE8) line. The libraries had 40,655,986–58,533,428 clean reads, and 95.00–96.50% reads were successfully mapped to the tobacco genome. Comparative analysis of the leaf transcriptome identified a total of 8,147 differentially expressed genes (DEGs) between the wild-type and OE8, of which 4,203 genes were upregulated, and 3,944 genes were downregulated (Supplementary Table 4). The PCA and cluster dendrogram of the transcriptome also supported the classification of gene expression patterns into two groups (Supplementary Figure 6A).

The DEGs were annotated to 44 GO terms (Supplementary Figure 6C), among which the three categories with the largest number of DEGs included “metabolic process” (1,148 genes), “catalytic activity” (1,074 genes), and “cell” (568 genes). KEGG pathway and enrichment analysis showed that DEGs were significantly assigned to 130 enriched pathways. The top five pathways in order of smallest to largest *q* value were “biosynthesis of secondary metabolites,” “metabolic pathways,” “MAPK signaling pathway,” “phenylalanine metabolism” and “flavonoid biosynthesis.” 664, 1,015, 114, 45, and 46 related genes were enriched in these pathways, respectively (Supplementary Figure 6D).

In the plant flavonoid-related pathway there were 46 DEGs. These include key enzyme-coding structural genes involved in flavonoid biosynthesis such as six phenylalanine ammonia lyases (PALs), one cinnamate 4-hydroxylases (C4Hs), six 4-coumarate-CoA ligases (4CLs), four chalcone synthases (CHSs), four chalcone isomerases (CHIs), two flavanone-3-hydroxylases (F3Hs), two flavonol synthase (FLSs), three flavonoid 3′-hydroxylases (F3’Hs), two dihydroflavonol reductases (DFRs) (Supplementary Figure 7). In addition, 343 transcription factors were found in the DEGs, of which 202 were upregulated and 141 were downregulated in the OE8. A total of 33 DEGs were found in the bHLH family, followed by 25 DEGs in the MYB family.

To test the reliability of transcriptome data, RT-qPCR assays were performed on six flavonoid structural genes that were differentially expressed in OE8. Gene expression as elucidated by RT-qPCR exhibited similar trends to that of the transcriptome data. RT-qPCR analysis showed that the expression of the flavonoid biosynthesis-related genes *FLS2*, *FLS1, DFR*, *F3H*, *CHI*, and *CHS* was upregulated in the OE8 lines compared to the control (Figure 4D). These results indicated that *EbMYBP1* positively regulates flavonoid biosynthesis, and *EbMYBP1* regulates genes expression directly or indirectly in the flavonoid synthesis pathway.

### *EbMYBP1* activated the transcription of flavonoid biosynthesis-associated genes

**FIGURE 5:**
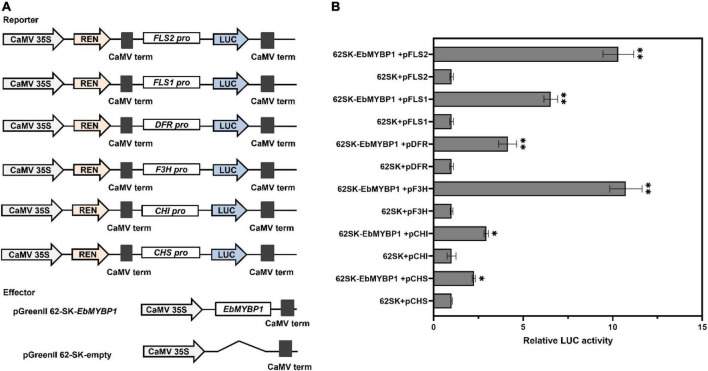
EbMYBP1 activated the expression of flavonoid–biosynthesis genes. **(A)** Diagrams of the reporter and effector constructs used in the dual-luciferase reporter assay. **(B)** Transient dual-luciferase reporter assays show that the activation of *FLS2, FLS1, DFR, F3H, CHI*, and *CHS* expression is activated by EbMYBP1. Transient expression assays used the promoter fragments of the *FLS2, FLS1, DFR, F3H, CHI*, and *CHS* genes. The luciferase luminescence intensities were quantitated following transfection with different vectors: 62SK represents empty pGreenII 62-SKvector; EbMYBP1 represents the pGreenII 62-SK-EbMYBP1 vector; *FLS2, FLS1, DFR, F3H, CHI*, and *CHS* represent pGreenII 0800-LUC-FLS2, pGreenII 0800-LUC-FLS1, pGreenII 0800-LUC-DFR, pGreenII 0800-LUC-F3H, pGreenII 0800-LUC-CHI, and pGreenII 0800-LUC-CHS vectors, respectively. Renilla luciferase (REN) was used for normalization. Data represent mean ± SD of three biological replicates. Asterisks indicate significant differences (*P < 0.05; ^**^P < 0.01).

To determine whether *EbMYBP1* directly regulate flavonoid biosynthesis related genes, we analyzed the cis*-*elements of the above six structural gene promoters. These promoter sequences were cloned, containing at least one conserved MYB-recognition element, indicating that *EbMYBP1* can bind to their promoters. We then investigate whether EbMYBP1 directly affects the transcriptional activity of the promoters of the six representative genes using a transient transactivation assay in tobacco leaves. The *cis*-elements, bound to MYB proteins within the promoter region of these six genes, were identified using PlantCARE. MYB-binding elements were found in the promoters of all six genes involved in flavonoid biosynthesis pathway (Supplementary Figure 8). Therefore, dual-LUC assays were performed to identify whether EbMYBP1 activates the promoters of these genes *in vivo*. The LUC activity levels from the *pGreen 0800:FLS2, pGreen 0800:F3H, pGreen 0800:FLS1, pGreen 0800:DFR, pGreen 0800:CHI, and pGreen 0800:CHS* reporters were 10.31-, 10.73-, 6.54-, 4.14-, 2.94-, and 2.24-fold higher in EbMYBP1 than in the presence of the control, respectively (Figure 5).

**FIGURE 6:**
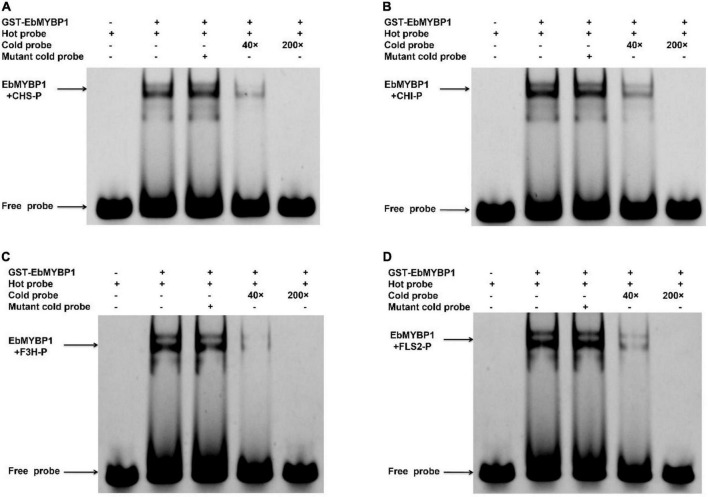
Electrophoretic mobility shift assay (EMSA) of EbMYBP1 binding to the CHS **(A)**, CHI **(B)**, F3H **(C),** and FLS2 **(D)** promoter region harboring EbMYBP1-binding sites *in vitro*.

To further investigate if EbMYBP1 bind to the MYB-binding *cis*-elements in the *FLS2, F3H, CHI*, and *CHS* promoters, EMSA was performed, the results of which confirmed that EbMYBP1 binds to their promoter regions (Figure 6). These findings indicate that EbMYBP1 activates *FLS2, F3H, CHI*, and *CHS* expression both *in vivo* and *in vitro*, possibly by binding directly to the *cis*-element of their promoters.

## Discussion

Flavonoids and flavonoid glycosides are abundant active components in *E. breviscapus* (Wu et al., 2021). However, the regulatory mechanism underlying flavonoid and flavonoid glycoside metabolism remains unknown. Here, a novel R2R3-MYB transcription factor was identified in *E. breviscapus*, revealing the highly conserved R2R3 domain typical of MYB transcription factors at its N-terminus (Figure 1A). Similar to the AtMYB11, AtMYB12, and AtMYB111 from *Arabidopsis* and MYB protein P from maize, EbMYBP1 does not contain the conserved motif for interacting with bHLH protein (Grotewold et al., 1994; Mehrtens et al., 2005; Stracke et al., 2007). Additionally, EbMYBP1 has the SG7-1 and SG7-2 motifs, which are employed as identifiers for transcription factors related to flavonoid production (Stracke et al., 2001; Czemmel et al., 2009; Wang et al., 2022), clustering with proteins as flavonoid-specific activators. Many studies have demonstrated that TFs in the same subgroup shares similar functions (Pandey et al., 2014).

Thus, we hypothesized that *EbMYBP1* also participates in the flavonoid metabolic pathway in *E. breviscapus*. Our findings suggested that the *EbMYBP1* expression level correlated with the accumulation of flavonoids in different tissues in *E. breviscapus*. Our results showed that scutellarin are mainly abundant in the leaves of *E. breviscapus* (Figure 2C). The expression pattern of *EbMYBP1* analysis revealed a correlation between *EbMYBP1* expression and scutellarin accumulation in different tissues, indicating that *EbMYBP1* may play a key role in flavonoid biosynthesis pathway. To further elucidate the function of *EbMYBP1*, *EbMYBP1* was transformed and overexpressed in tobacco via the agrobacterium method.

The metabolome analysis showed that *EbMYBP1* overexpression in tobacco resulted in significant flavonoid accumulation. This indicates that *EbMYBP1* is involved in the regulation flavonoid biosynthesis in leaves compared to the WT. Meanwhile, the total flavonoids content was significantly higher in transgenic tobacco plants than that of WT (Figure 4B). The reason might be that the overexpression of *EbMYBP1* significantly enhanced those genes expression involved in flavonoid biosynthesis. Therefore, the flavonoid metabolism has been expanded, resulting in increasing flavonoid accumulation. Additionally, flavonols are synthesized in all organs, however, other active components like anthocyanins, are accumulated in specific cell types (Tan et al., 2019). Consequently, there is a potential competition between flavonols and anthocyanins. Our results showed that the accumulation of kaempferol and its derivatives was increased.

In this study, the metabolome analysis identified 98 flavonoid-related metabolites. A dramatically higher level of 3,7-Di-O-methylquercetin and kaempferol 3-O-rutinoside, which were the flavonol metabolites that differed significantly, were found in leaves of OE8 compared to those in WT plants (Figure 2). Meanwhile, increased amounts of anthocyanin 3-O-beta-D-glucoside and cyanidin 3-O-galactoside in anthocyanin were detected within the leaves of OE8 relative to the WT (Supplementary Table 2). Previous studies showed that in *Gerbera hybrida*, *GhMYB1a* overexpression activates *FLS* expression, causing an increased in kaempferol and decrease in anthocyanins (Zhong et al., 2020). Overexpression of *PpMYB15* or *PpMYBF1* from *Prunus persica* L. Batsch led to significant accumulation of quercetin and kaempferol in their transgenic tobacco flowers (Cao et al., 2019). Similar results showed that overexpression of *AtMYB12* (Luo et al., 2008; Misra et al., 2010), *GtMYBP4* (Nakatsuka et al., 2012), *AtMYB11* (Pandey et al., 2015), *EsMYBF1* (Huang et al., 2016), and *CcMYB12* (Blanco et al., 2018) led to accumulation of quercetin and kaempferol levels in transgenic tobacco flowers. Moreover, overexpression of grape *MYBF1* or apple *MYB22* complemented the deficient phenotype in flavonols in an *Arabidopsis* mutant (Czemmel et al., 2009; Wang et al., 2017).

Subsequently, transcriptomic analysis indicated that many genes related to flavonoid biosynthesis, from *PAL* to *ANS* genes, could be either directly or indirectly up-regulated by *EbMYBP1*. Moreover, the transcript abundances of the structural genes involved in flavonoid biosynthesis coincided with corresponding higher accumulated metabolite levels detected (Figure 3). RT-qPCR analysis of six flavonoid biosynthesis-related genes (*FLS2*, *FLS1*, *CHI*, *F3H*, *CHS*, and *DFR*) was further performed in transgenic tobacco. The RT-qPCR results showed that the expression levels of *FLS2*, *FLS1*, *CHI*, *CHS*, and *F3H* were significantly upregulated. Furthermore, *DFR* was also upregulated to a certain extent compared with the levels in WT. Previous studies showed that overexpression of the MYB gene not only upregulated expression of *PAL*, *CHS*, and *CHI*, but also anthocyanin biosynthesis genes such as *DFR* in the tobacco plants over-expressing *EsMYB90* (Qi et al., 2020). In previous studies, it has been demonstrated that many R2R3-MYB transcription factors regulate flavonoid biosynthesis by interacting with the promoters of the targeted structural genes (Wang et al., 2017). The sequence of gene promoters also varies greatly in different plant species. Thus, the MYB TFs might exhibit different regulatory functions in flavonoid biosynthesis in different species (Liu et al., 2016).

Previous studies showed that *AtMYB12* is involved in flavonoid biosynthesis and regulates the expression of *AtCHS*, *AtCHI*, *AtF3H*, and *AtFLS* (Mehrtens et al., 2005; Czemmel et al., 2009; Stracke et al., 2010). It has been demonstrated that upregulation of these genes gives rise to increased flavonoid accumulation in *Arabidopsis* (Guo et al., 2015; Wang et al., 2016, 2018). Flavonol synthase (*FLS*) and dihydroflavonol4-reductase (*DFR*) generally target the same substrate dihydroflavonol, leading to competition between flavonol and anthocyanin (Davies and Schwinn, 2003). It was found that strong upregulation of *NtFLS* and *NtF3H* may be crucial in promoting the branch of flavonol biosynthesis, whereas weak upregulation of *NtDFR* would decrease the branch of the anthocyanin biosynthesis. Moreover, *CHS, CHI, F3H*, and *FLS* genes are related to flavonoid biosynthesis, primarily being activated by the SG7 MYB factors like *MYB11*, *MYB12*, and *MYB111*, although SG7 MYB-independent flavonol biosynthesis was reported in pollen grains and siliques (Stracke et al., 2010). In this study, *EbMYBP1* overexpression also activated the expression of *NtDFR*. Of these, DFR is related to anthocyanin and proanthocyanidin biosynthesis, and anthocyanidins are synthesized by ANS from leucoanthocyanidin produced by DFR. Meanwhile, EbMYBP1 activated the promoter of *NtDRF*, the gene that is thought to underlie anthocyanin synthesis.

In the flavonoid biosynthesis pathway, structural genes such as *CHS*, *CHI, F3H*, *FLS2*, *FLS1*, and *DFR* participated in flavonoid biosynthesis. Multiple cis-acting element were identified in these promoter regions, including MYB-binding elements, light-responsive elements, hormone-responsive elements, and elements for low-temperature responses (Supplementary Figure 8). These analyses indicated that various environmental and genetic factors may affect the biosynthesis of flavonoids. Thus, the promoters of these flavonoid biosynthesis genes were selected as potential targets of EbMYBP1 transcription activation. Dual-luciferase assay indicated that EbMYBP1 could bind and significantly activate the expression of *FLS2*, *F3H*, and *FLS1*, rather than *DFR, CHI*, and *CHS* (Figure 5). A similar promoter assay result was observed for *AtMYB12/11/111*, which was capable of activating *CHS*, *CHI*, *F3H*, and *FLS* promoters as well (Mehrtens et al., 2005; Stracke et al., 2007). Nevertheless, both *VvMYBF1* and *MdMYB22* have been shown to activate the promoters of *VvCHI*, *VvFLS*, and *MdFLS* in grapes and apples, respectively (Czemmel et al., 2009; Wang et al., 2017). Transient tobacco expression studies have reported that *CsMYBF1* can only activate the *CsCHS* and *CsFLS* promoters (Liu et al., 2016). The EMSA assay showed that EbMYBP1 could specifically activate the expression of *FLS2, F3H, CHI*, and *CHS* genes, which function in flavonoid biosynthesis, leading to high levels of flavonoid production.

Based on our present findings, *EbMYBP1*, could regulate flavonoid biosynthesis by directly activating the expression of *FLS2*, *CHI*, *CHS*, and *F3H* genes. In conclusion, we identified a transcriptional activator *EbMYBP1* that is positively involved in flavonoid biosynthesis by directly activating flavonoid-related genes. The findings reported here expanded our understanding of the intricate transcriptional regulatory network of flavonoid biosynthesis.

## Data availability statement

The datasets presented in this study can be found in online repositories. The names of the repository/repositories and accession number(s) can be found in the article/Supplementary material.

## Author contributions

YZ, CZ, and SY conceived, designed the experiments, and wrote the article. YZ, QT, and QG performed the experiments. GZ, WS, GX, XL, GL, WF, and XL analyzed the data. All authors contributed to the article and approved the submitted version.
