# Supplementary material for: EbMYBP1, a R2R3-MYB transcription factor, promotes flavonoid biosynthesis in Erigeron breviscapus
Source: Front Plant Sci. 2022 Jul 28;13:946827. doi: 10.3389/fpls.2022.946827 (PMC9366350; doi:10.3389/fpls.2022.946827)
Supplement: Supplementary file 1 [file Data_Sheet_1.docx]

**Supplementary Data**


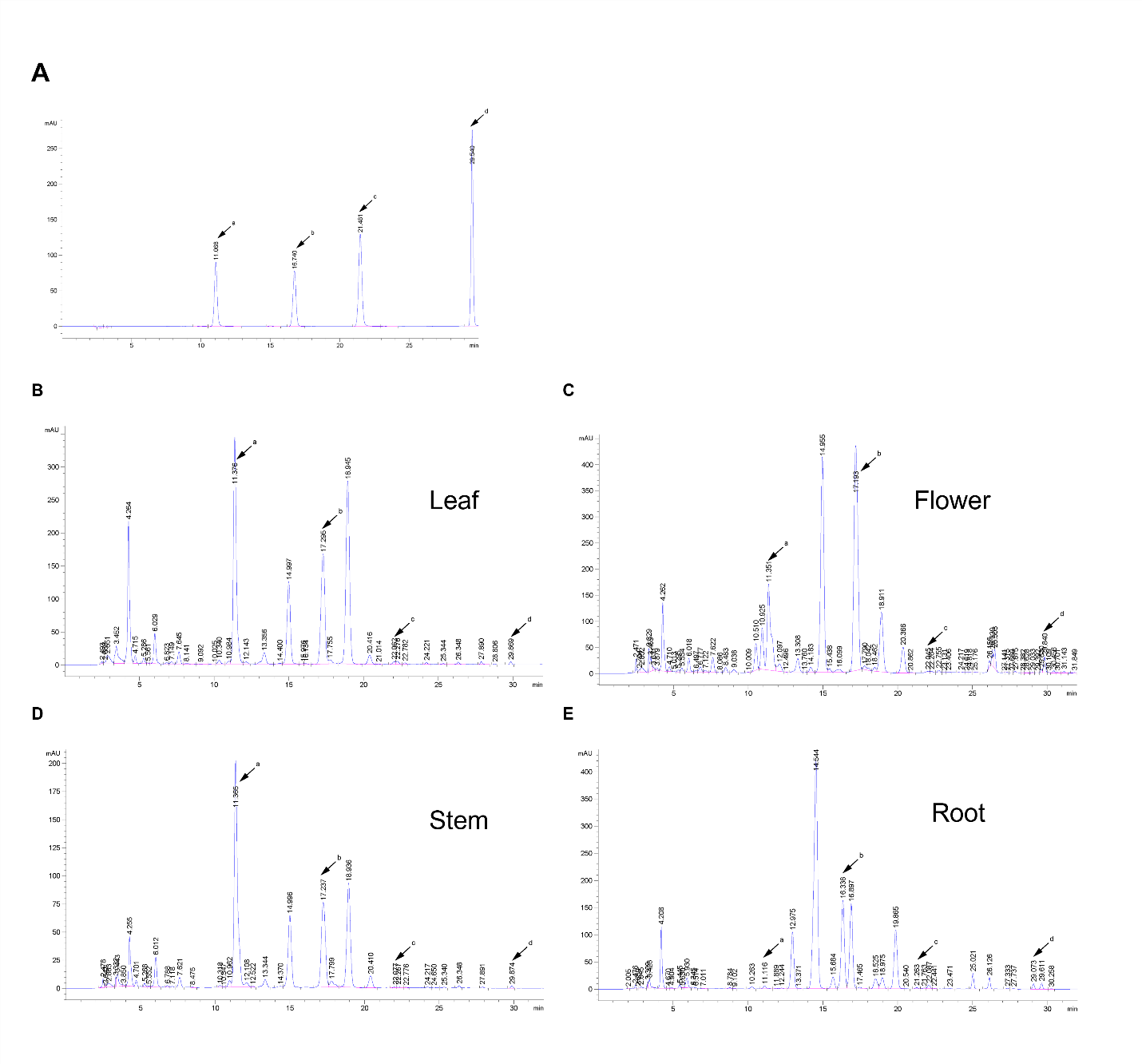


**Figure S1. HPLC chromatograph of active components from different tissues in *E.breviscapus*.** (A) Standard samples. (B) The leaf of *E.breviscapus*. (C) The Flower of *E.breviscapus*. (D) The stem of *E.breviscapus*. (E) The root of *E.breviscapus*. Scutellarin(a), Apigenin-7-O-glucuronide(b), Scutellarein(c), Apigenin(d).


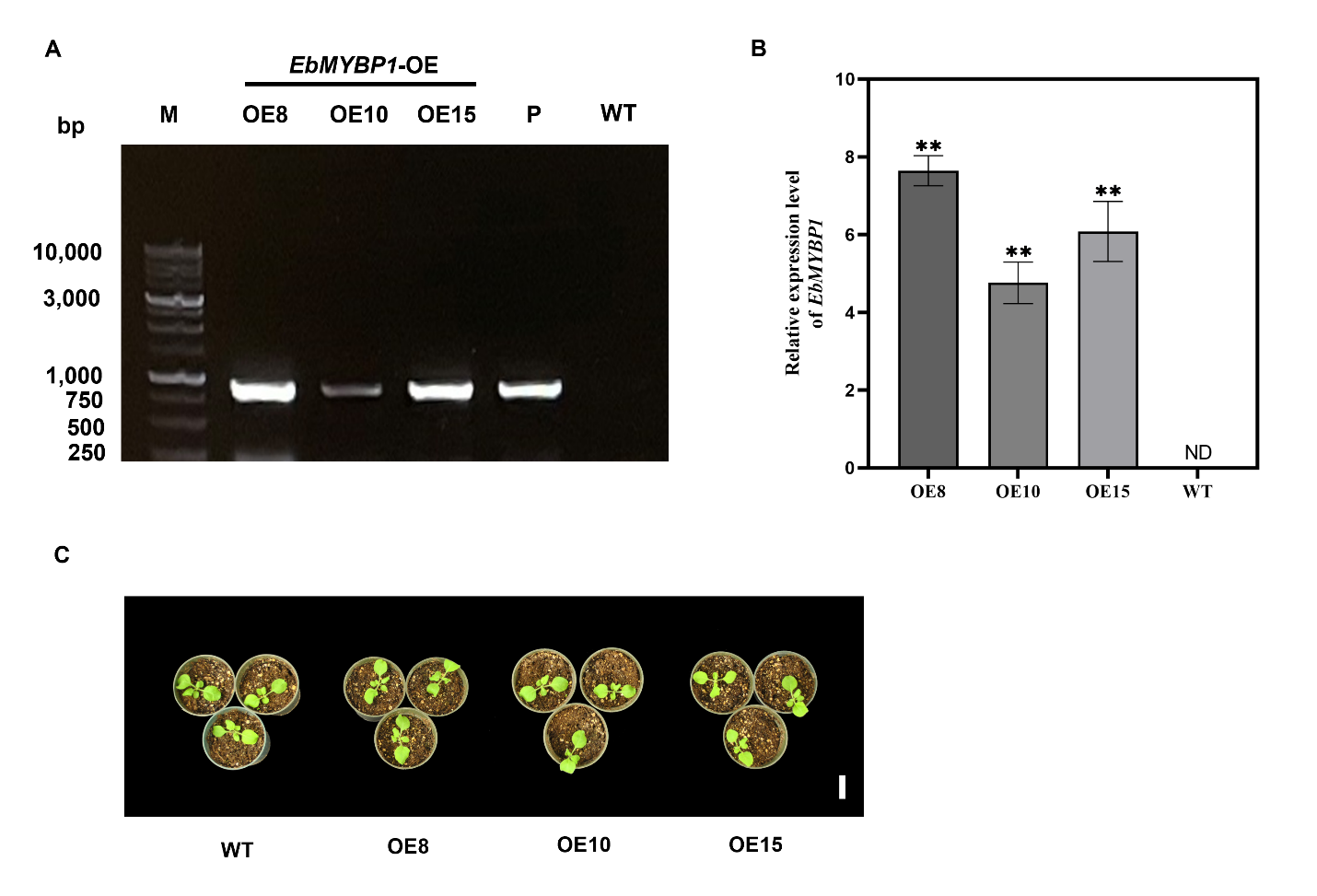


**Figure S2. Characterization of overexpression *EbMYBP1* transgenic tobacco lines.**

(A) PCR amplification of *EbMYBP1* in transgenic lines (OE8, OE10 and OE15) and WT type plants; Plasmid is as positive control (P).

(B) Relative expression of *EbMYBP1* in transgenic lines (OE8, OE10 and OE15) and WT type plants using RT-qPCR.

(C) Phenotypic analysis of *EbMYBP1* transgenic tobacco plants. Bar=1cm.


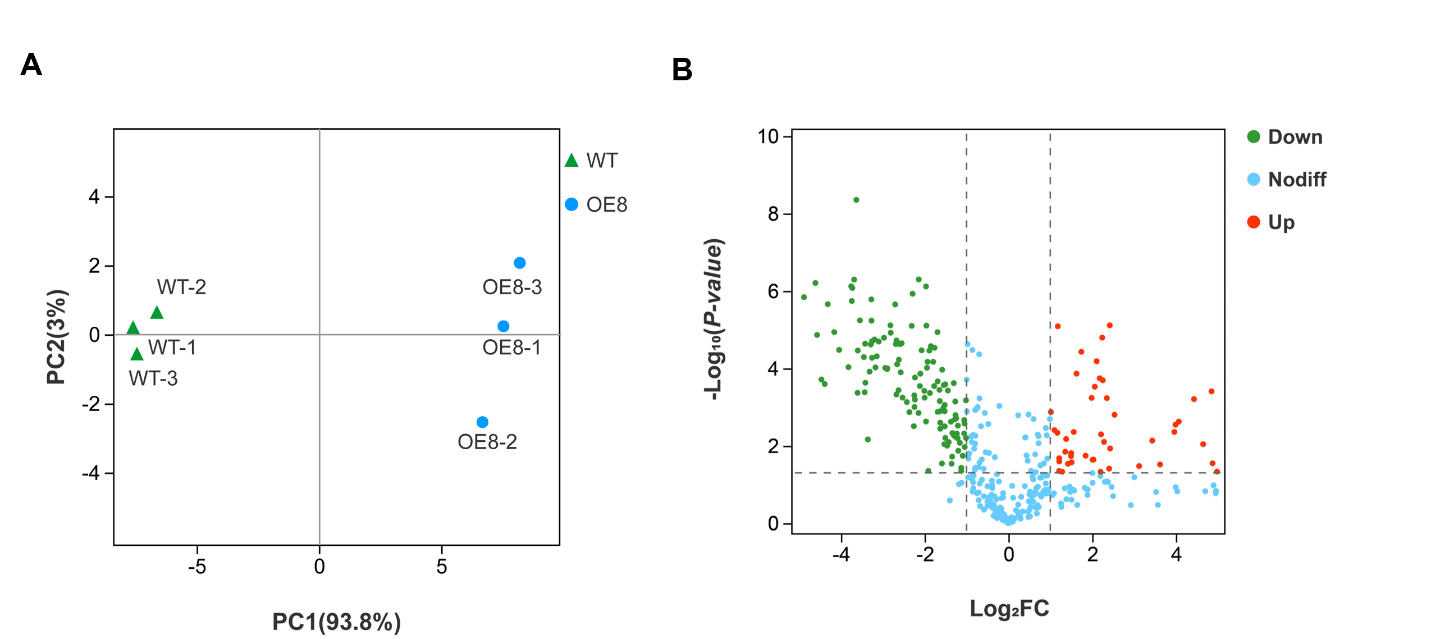


**Figure S3. Metabolome profiling of the leaves of WT and OE8.**

(A) Principal component analysis (PCA) of metabolome data from leaves of WT and OE8.

(B) Volcano plots of the leaves of WT and OE8 metabolome profiles. Volcano plots show the log 2-fold changes and the − log10 adjusted p-values in metabolite. Data were selected at the cut off values adj-p < 0.05 and fold change > 2. Red and green dots represent metabolites having their level significantly altered.


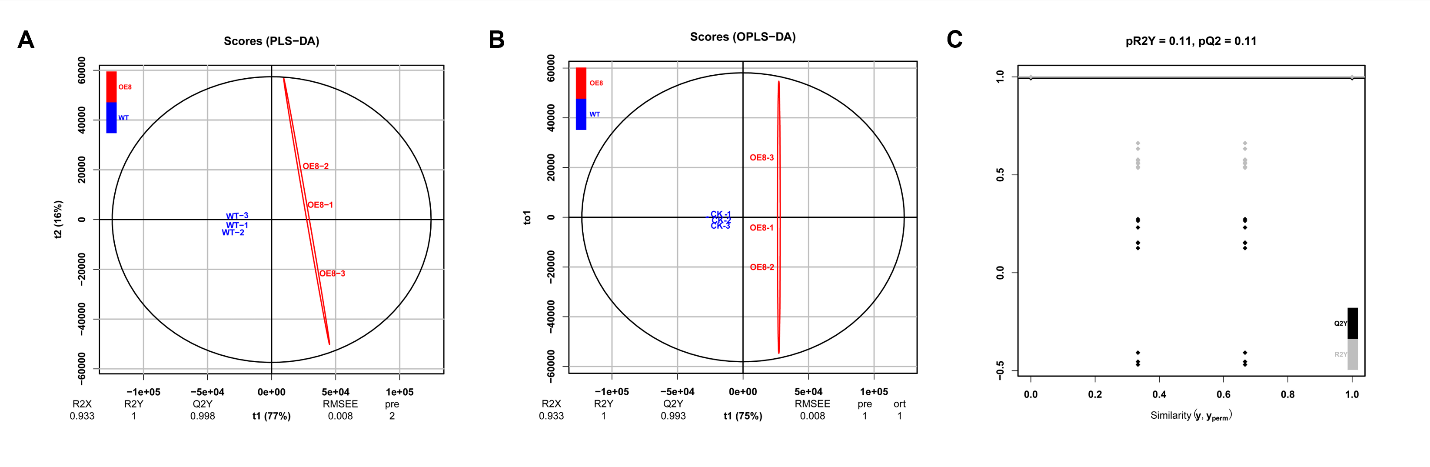


**Figure S4 Phenotypic and multivariate statistical analysis of WT and OE8.**

(A) Partial least squares discriminant analysis, (B) Orthogonal projection to latent structures-discriminant analysis, (C) Model validation diagram.


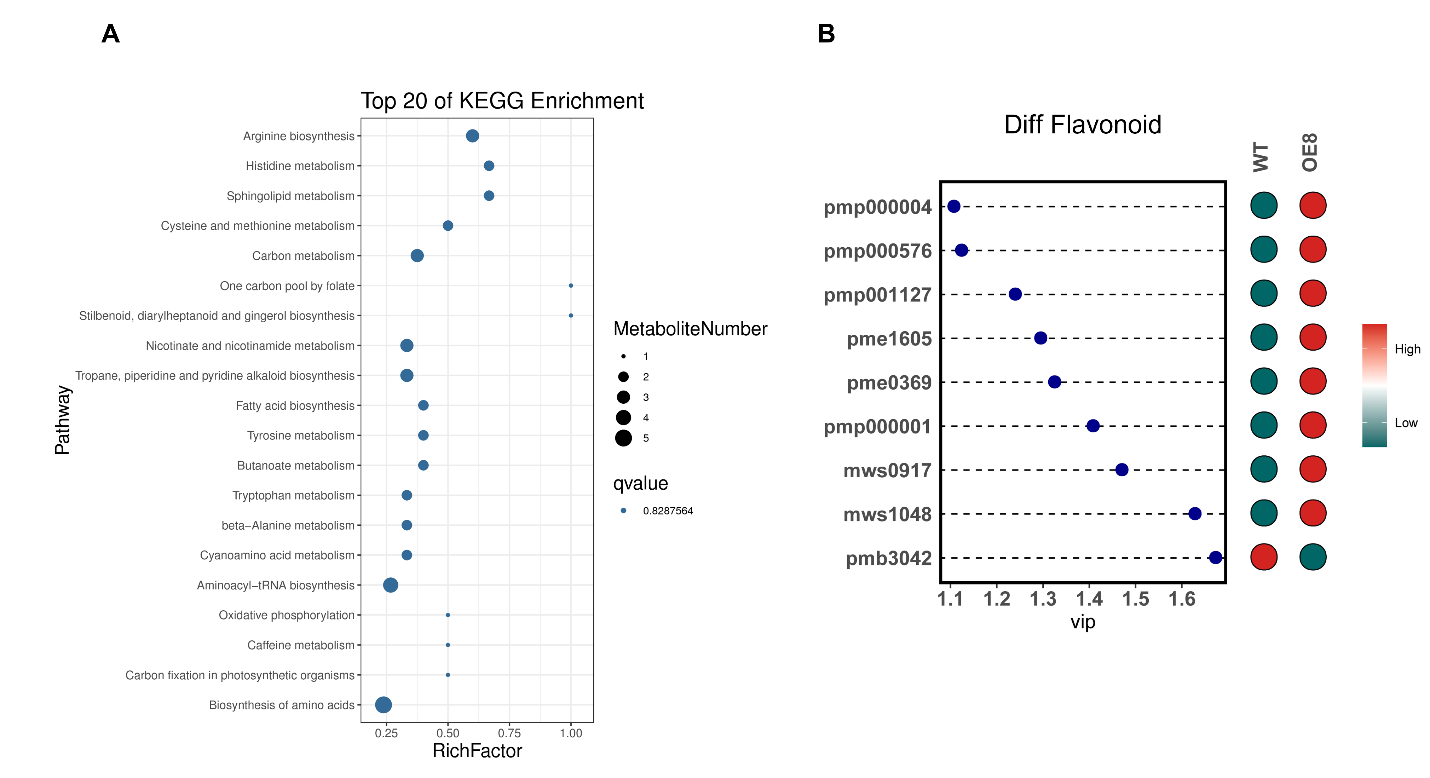


**Figure S5 Metabolome differences in leaves between WT and OE8.**

(A) KEGG analysis illustrates the enrichment pathway for the significantly differential expressed metabolites. (B) Top nine differentially accumulated flavonoid.


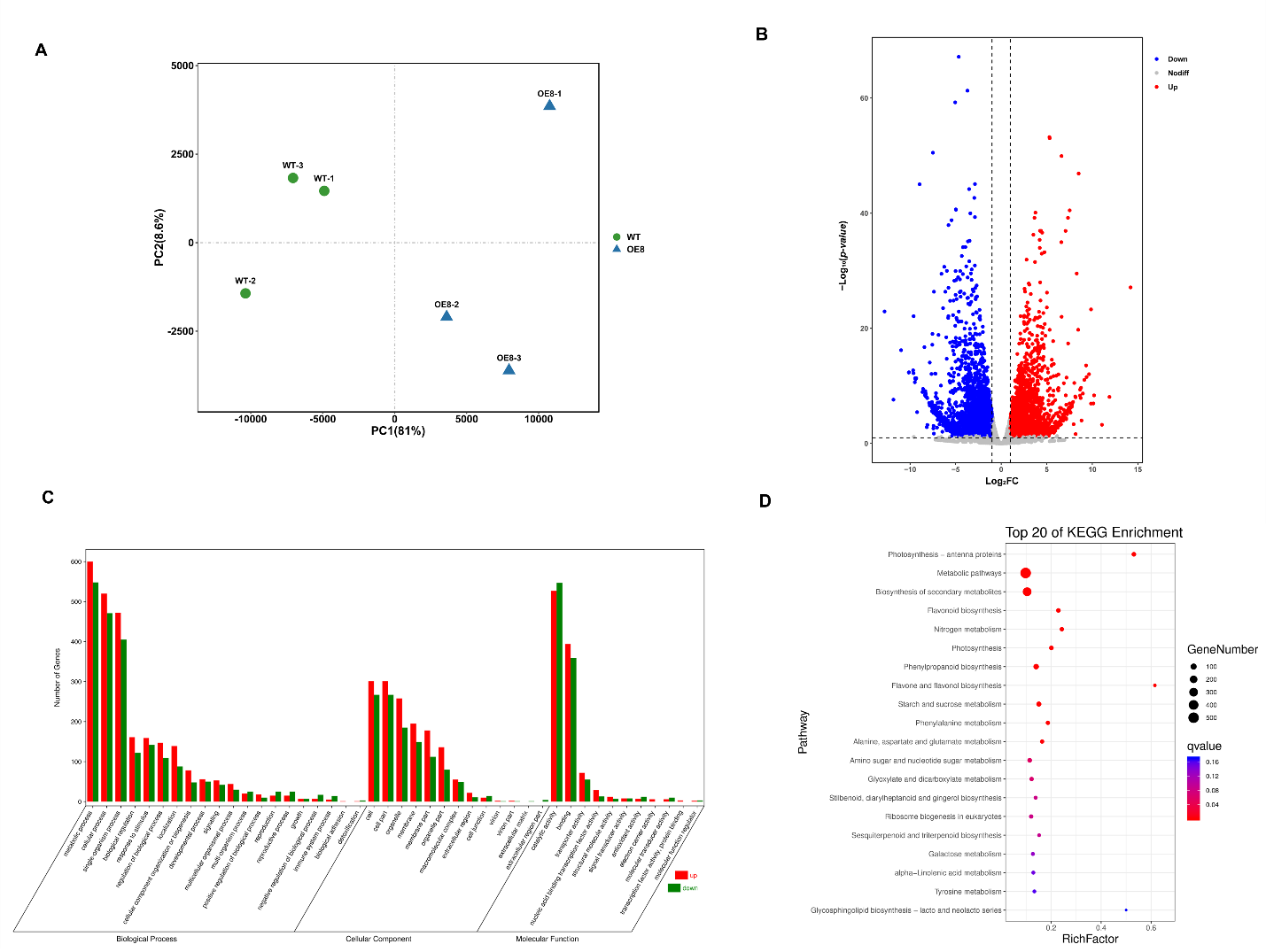


**Figure S6. Transcriptome profiling of the leaves of WT and OE8.**

(A) Principal component analysis (PCA) of transcriptome data from leaves of WT and OE8.

(B) Volcano plots of the leaves of WT and OE8 transcriptome profiles. Volcano plots show the log 2 fold changes and the − log10 adjusted p-values in transcripts. Data were selected at the cut off values adj-p < 0.05 and fold change > 2. Red and blue dots represent transcripts having their level significantly altered.  (C) GO terms analysis. (D) KEGG analysis.


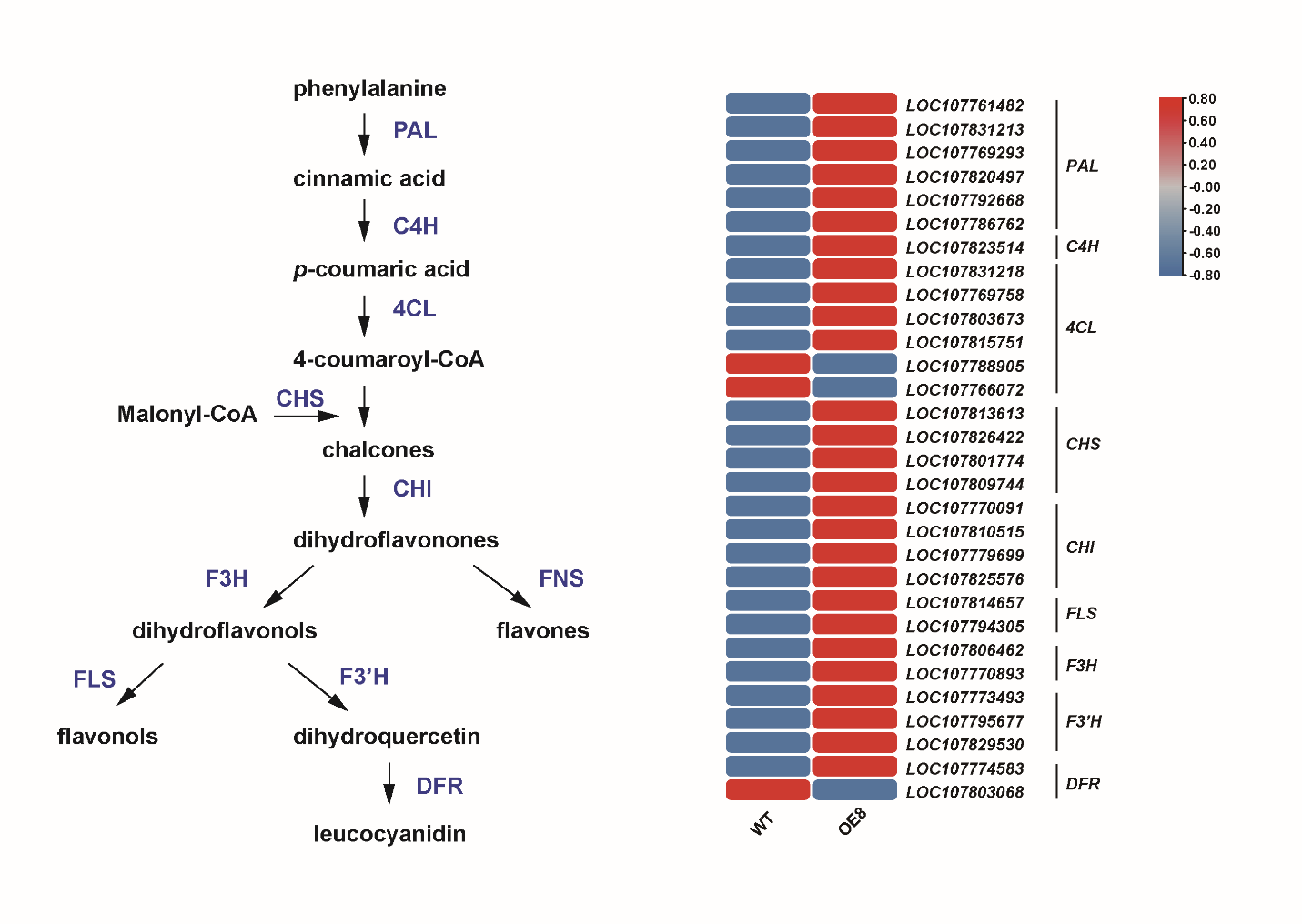


**Figure S7. Heat map representation of the expression patterns of flavonoid-related genes.** Gene expression is displayed as heat map depicting the log2 (FPKM) values. Red and blue font indicate up- and down-regulated genes, respectively.


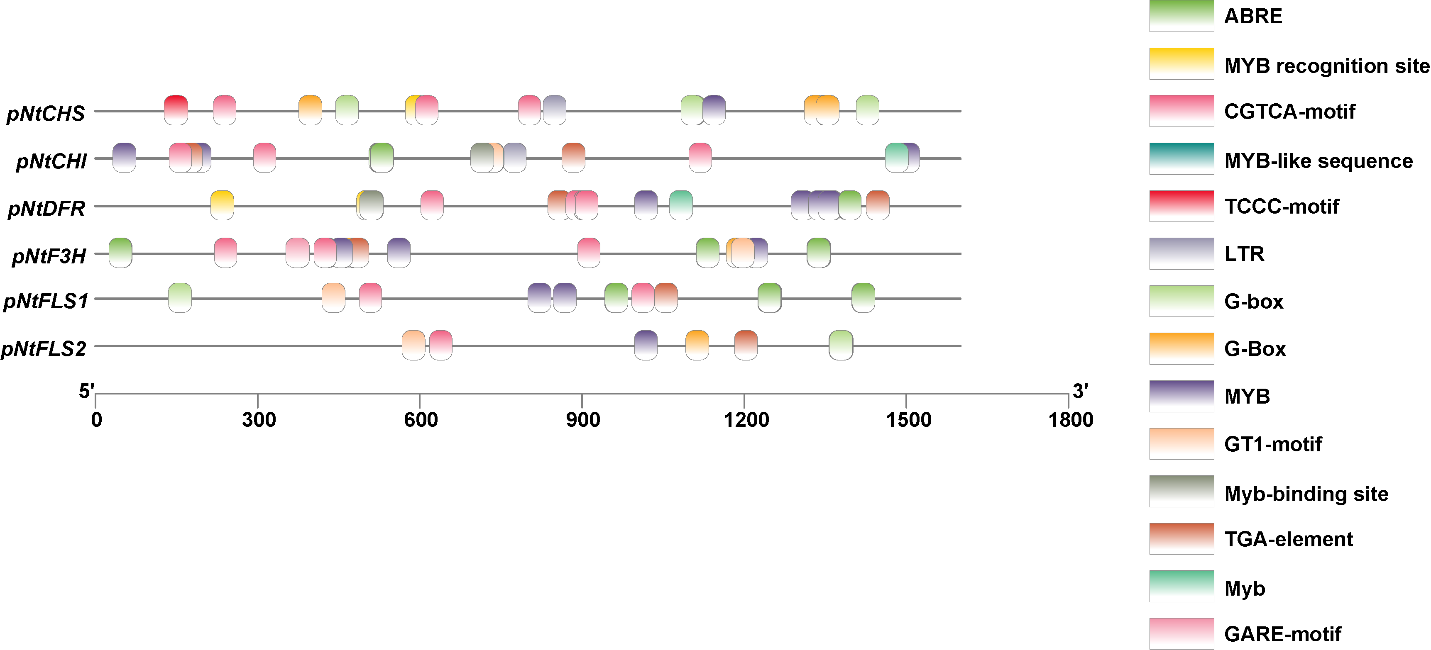


**Figure S8. Cis-acting regulatory elements in gene promoters.**

Cis-acting regulatory elements were analyzed by searching the PlantCARE database of (http://bioinf ormatics.psb.ugent.be/w ebtools/plantcare/html/) in the promoters of *NtCHS*, *NtCHI*, *NtDFR*, *NtF3H*, *NtFLS1* and *NtFLS2*.
