# Supplementary material for: EbMYBP1, a R2R3-MYB transcription factor, promotes flavonoid biosynthesis in Erigeron breviscapus
Source: Front Plant Sci. 2022 Jul 28;13:946827. doi: 10.3389/fpls.2022.946827 (PMC9366350; doi:10.3389/fpls.2022.946827)
Supplement: Supplementary file 2 [file Table_1.docx]

**Table S1. List of primers used in this study**

| **Primer** | **Sequence (5'-3')** | **Useage** |
| --- | --- | --- |
| EbMYBP1-F | CCCGGGATGGGAAGAGCGCCATGTTGTG | *EbMYBP1* cloning |
| EbMYBP1-R | TCTAGATCCGGATAGAAGCCATGCCTCTAG |  |
| qNtFLS2-F | AAAACTCCAGGGTCTCAG | RT-qPCR |
| qNtFLS2-R | CTGTAGGAGGGAGGATTT |  |
| qNtF3H-F | CCATAACATTTGCAGAGATGTAC |  |
| qNtF3H-R | TCTTCAATGGGCTTGGTTTTCA |  |
| qNtCHS-F | GGCTCCGTCCATCGGTCAA |  |
| qNtCHS-R | TCATTGGGTCCACGAAAC |  |
| qNtCHI-F | TCTCGCCGCTAAATGGAA |  |
| qNtCHI-R | TACCCGTCAAAGGCAAGA |  |
| qNtDFR-F | CTAGCTTAATCACTGCCCTTTCA |  |
| qNtDFR-R | TAGTACTCCGGCCATTTCTCTT |  |
| qNtFLS1-F | AAGACTCCAGGGTCGCAG |  |
| qNtFLS1-R | CTGTAGGAAGGAGGGCTT |  |
| qNtactin-F | CTGAGGTCCTTTTCCAACCA |  |
| qNtactin-R | TACCCGGGAACATGGTAGAG |  |
| EbMYBP1-F | TAATGGACCCGAATGGCGAG |  |
| EbMYBP1-R | TTCCCACCCCCTAATCCGAA |  |
| EbPAL-F | CATTGAACTGGGGAGTGGCT |  |
| EbPAL-R | ACTACTCGCTTTCACACCCG |  |
| EbCHS-F | ACATGCCTGGTGCTGACTAT |  |
| EbCHS-R | GAGCTTGTGCAACGAGTGAA |  |
| EbCHI-F | GAATGGAAAGGACGAACGGC |  |
| EbCHI-R | CAGCATGTTGGAGGGGTCAA |  |
| Ebactin-F | GAAAGTTGGGGGCTCGAAGA |  |
| Ebactin-R | TTCTCATAAGGTGCCAGCGG |  |
| EbMYBP1-F | ATGGGAAGAGCGCCATGTTGTG | Identification of OE plants |
| EbMYBP1-R | GTCGTAGCACCATTCCATGGCC |  |
| 35S-MYBP1-EcoRIF | CAAGAGACAGGATCCGAATTCATGGGAAGAGCGCCATGTTG | Subcellular localization |
| 35S-MYBP1-SalIR | CATCGGTGCACTAGTGTCGACGGATAGAAGCCATGCCTCTAG |  |
| 35S-MYBP1-TSalIR | CATCGGTGCACTAGTGTCGACTCAGGATAGAAGCCATGCCTC |  |
| 62SK-EbMYBP1-EcoRIF | TCCCCCGGGCTGCAGGAATTCATGGGAAGAGCGCCATGTTG | Dual-LUC |
| 62SK-EbMYBP1-SalIR | GGGCCCCCCCTCGAGGTCGACTCAGGATAGAAGCCATGCCT |  |
| 0800-pNtCHS-HindIIIF | GTCGACGGTATCGATAAGCTTATTATTTATTTCTGTGTCTG |  |
| 0800-pNtCHS-BamHIR | CGCTCTAGAACTAGTGGATCCTTTCGCCGGAAAAAATGATG |  |
| 0800-pNtCHI-HindIIIF | GTCGACGGTATCGATAAGCTTTGAAGGGATGCTCCTCGTCCTA |  |
| 0800-pNtCHI-BamHIR | CGCTCTAGAACTAGTGGATCCTGCTCTCTACTTTTTGATTTCA |  |
| 0800-pNtF3H-KpnIF | CTATAGGGCGAATTGGGTACCAAAAAGTGATTTAGGAGAGA |  |
| 0800-pNtF3H-SalIR | AAGCTTATCGATACCGTCGACTTTTAGTAATCTTCCTCGCT |  |
| 0800-pNtDFR-HindIIIF | GTCGACGGTATCGATAAGCTTAACTAACGATTTGCCTTGTCAT |  |
| 0800-pNtDFR-BamHIR | CGCTCTAGAACTAGTGGATCCTTTCAGAAATGAAAGGTAGAAG |  |
| 0800-pNtFLS1-HindIIIF | GTCGACGGTATCGATAAGCTTACTGCCAGAAATCGGAATCGAA |  |
| 0800-pNtFLS1-BamHIR | CGCTCTAGAACTAGTGGATCCTTTTCTTCTTAAGACCCTTAAT |  |
| 0800-pNtFLS2-HindIIIF | GTCGACGGTATCGATAAGCTTAAAGTTCTTATTTTATTGTTGT |  |
| 0800-pNtFLS2-BamHIR | CGCTCTAGAACTAGTGGATCCTTTTTTCTTAAGATTTCCGTTA |  |
| 4T-EbMYBP1-EcoRIF | CCGCGTGGATCCCCGGAATTCATGGGAAGAGCGCCATGTTG | EMSA |
| 4T-EbMYBP1-SalIR | GATGCGGCCGCTCGAGTCGACTCAGGATAGAAGCCATGCCT |  |
| pNtCHS-FF | GATCTTGAGAAGTAGGTAGCTAGCTAATTATTG |  |
| pNtCHS-CF | GATCTTGAGAAGTAGGTAGCTAGCTAATTATTG |  |
| pNtCHS-R | CAATAATTAGCTAGCTACCTACTTCTCAAGATC |  |
| pNtCHS-MF | GATCTTGAGAAAAAAAAAGCTAGCTAATTATTG |  |
| pNtCHS-MR | CAATAATTAGCTAGCTTTTTTTTTCTCAAGATC |  |
| pNtCHI-FF | CCTCTCGCTCTACCTAACTACAAATTAT |  |
| pNtCHI-CF | CCTCTCGCTCTACCTAACTACAAATTAT |  |
| pNtCHI-R | ATAATTTGTAGTTAGGTAGAGCGAGAGG |  |
| pNtCHI-MF | CCTCTCGCTCTAAAAAAATACAAATTAT |  |
| pNtCHI-MR | ATAATTTGTATTTTTTTAGAGCGAGAGG |  |
| pNtF3H-FF | CAGGGTTAAAGTTAGTTGTAAGGAGAGG |  |
| pNtF3H-CF | CAGGGTTAAAGTTAGTTGTAAGGAGAGG |  |
| pNtF3H-R | CCTCTCCTTACAACTAACTTTAACCCTG |  |
| pNtF3H-MF | CAGGGTTAAAGAAAAAAATAAGGAGAGG |  |
| pNtF3H-MR | CCTCTCCTTATTTTTTTCTTTAACCCTG |  |
| pNtFLS2-FF | GAAAAAGTGAAGTTTGGTAGGTAGTATTAATGAA |  |
| pNtFLS2-CF | GAAAAAGTGAAGTTTGGTAGGTAGTATTAATGAA |  |
| pNtFLS2-R | TTCATTAATACTACCTACCAAACTTCACTTTTTC |  |
| pNtFLS2-MF | GAAAAAGTGAAGTTTAAAAAAAAGTATTAATGAA |  |
| pNtFLS2-MR | TTCATTAATACTTTTTTTTAAACTTCACTTTTTC |  |
